# Sample preparation of some plant tissues for transmission electron microscopy using high pressure freezing and freeze substitution

**DOI:** 10.1186/s40529-026-00499-x

**Published:** 2026-10-15

**Authors:** Chia-Mei Hsu, Yi-Chia Chou, Hsiao-Jung Yen, Wann-Neng Jane

**Affiliations:** Institute of Plant and Microbial Biology, Academia Sinica, No. 128, Sec. 2, Academia Road, Nankang, Taipei 11529 Taiwan

**Keywords:** High pressure freezing, Freeze substitution, Ultrastructure, Root, Anther, Leaf, Orchid petal

## Abstract

For sample preparation of biological block sectioning, high pressure freezing and freeze substitution (HPF-FS) is a better choice than chemical fixation. Freezing fixation can retain the original structures and preserve the soluble materials, and avoid the disadvantage of chemical fixation. Using high-pressure freezing, plant tissues are more difficult to freeze successfully than animal tissues and microbes because they have cell walls and varying degrees of vacuolation. For plant tissues, including the roots of *Arabidopsis thaliana*, 1-hexadecene is an ideal cryoprotectant/filler. The ability of highly vacuolated plant tissues (such as mature leaves and petals) to effectively remove intercellular air is crucial for successful high pressure freezing. Using the anthers of *Arabidopsis thaliana*, the leaves of *Brachypodium distachyon*, *Axonopus compressus* and *Plantago asiatica*, and the petals of *Phalaenopsis bellina* as examples, we found an effective method to remove air from the intercellular spaces: plant tissue in 1-hexadecene is placed in a vacuum desiccator, and then the air is removed using a vacuum pump, resulting in the release of numerous air bubbles. Plant tissue samples taken at different degassing times are then subjected to high pressure freezing and the subsequent required procedures of sample preparation. Based on the presentation of fine structures, the optimal time for different plant tissues can be determined and shows a positive correction with sample thickness. Good HPF-FS results show that the lipid bilayer within each organelle of the plant cells is clearly visible.

**Supplementary Information:**

The online version contains supplementary material available at https://doi.org/10.1186/s40529-026-00499-x.

## Introduction

High pressure freezing combined with freeze substitution (HPF-FS) is the best choice for biological sample preparation of transmission electron microscopy for block sectioning which can be used in morphology or immunolabeling. Freezing fixation (physical fixation) can retain the original structures and preserve the soluble materials, and avoid the disadvantage of chemical fixation including change of morphology and loss of cell contents.

Sample preparation of transmission electron microscopy using HPF-FS is a widely used method and has good results in animals, but is less frequently used for plants. Compared to animals, high vacuolation is a common characteristic of plants, especially in mature tissue, for example leaves and roots. The effect of cryofixation depends on the concentration of the sample solute and freezing rate, so the plants are more difficult to undergo cryofixation than the animals. Plant cells contain varying degrees of vacuolation, and more vacuoles are present in the mature tissues. The vacuoles are composed of water and soluble substances. The intercellular spaces are present in the plant tissues, and their main inclusions are water in roots and air in leaves.

When the plant tissues undergo high pressure freezing, some chemicals are used as cryoprotectant/filler, such as 1-Hexadecene (Morphew 2000), 0.15 M sucrose (Segui-Simarro, et al., 2004), 15% or 20% dextran/ 50 mM Tricine buffer (Shimoni et al. 2005; Kirchhoff et al. 2011; Bussi, et al. 2019) and 10% methanol + paste of yeast (Bobik et al. 2014). 1-Hexadecene is a hydrophobic substance with osmotic activity (Morphew 2000), and it can permeate into intercellular air space and is commonly used cryoprotectant/filler in plants, especially in leaves. However, air is always present in leaves.

Air is the most important factor to cryofixation and affects the freezing rate. The successful cryofixation depends on effective degas in plant leaves. We found a good method that small pieces of leaves in 1-hexadecene are degassed by pumping.

## Materials and methods

1.2 mm long root tips of *Arabidopsis thaliana* (14d seedling grown on agar plates) were loaded into 1.2mmx200µm carriers and filled with 1-hexadecene. Freezing samples were used by Leica EM PACT2 high pressure freezer under pressure 2020–2050 bar, and the freezing rate was over 10,000℉/s.

Leaves of *Brachypodium distachyon* and *Axonopus compressus* cut to 1mmx1mm size with knife, and leaves of *Plantago asiatica* and petals of *Phalaenopsis bellina* sampled by 1.2 mm-diameter sample punch, and flowers of *Arabidopsis thaliana* were transferred to beakers containing 1-Hexadecene. The sample-containing beakers in vacuum desiccator were pumped to 2 × 10^− 2^ torr and stayed 3 min, 5 min, 8 min. 10 min, 12 min, or 15 min.

Pumped-ready samples including anthers of *Arabidopsis thaliana* from flowers cut, and leaves of *Brachypodium distachyon*, *Axonopus compressus* and *Plantago asiatica* were put into 1.2mmx200µm carriers and filled with 1-hexadecene. Freezing samples were used by Leica EM PACT2 under pressure 2020–2050 bar, and freezing ratio was over 10,000℉/s.

Pumped-ready petals of *Phalaenopsis bellina* were transferred to slides and cut to two-halves by knife. A half containing upper epidermis put into 1.2mmx400µm carriers and upper epidermis was towards to carrier bottom. Freezing samples were used by Leica EM PACT2 under pressure 2020–2050 bar, and freezing ratio was over 10,000℉/s.

The freezing samples were transferred to -160℃ chamber (Leica AFS2 auto freeze substitution system) and stayed 5 min, and then chamber temperature raised to -90℃.

After removing as much freezing 1-hexadecene as possible from the sample surface, the freezing samples were transferred into substitution solution (2% OsO4/Acetone or 0.1%UA + 1%OsO4/Acetone for morphology; and 0.1%UA + 0.2%GA /Ethanol for immunolabeling). AFS protocol was followed: -90℃ 12 h; -90℃ → -80℃, 1℃/h; -80℃ 12 h; -80℃ → -70℃, 1℃/h; -70℃ 12 h; -70℃ → -60℃, 1℃/h; -60℃ 12 h; -60℃ → -20℃, 5℃/h; -20℃ 12 h; -20℃ → 0℃, 5℃/h; 0℃ 12 h; 0℃ → 20℃, 5℃/h; 20℃ 12 h.

The samples were rinsed with pure acetone or alcohol twice, 12 h each time. Embedding medium (Spurr’s resin or EMBed 812 resin for morphology; and LR Gold resin for immunolabeling) was used for infiltration and embedding. Concentration of infiltrating medium was 10%, 25%, 50%, 75%, 90%, 100%, 100%, and every step stayed 12–24 h. Polymerization of Spurr’s resin and EMBed 812 resin was at 70℃, 12 h and 60℃, 48 h, respectively. Polymerization of LR Gold resin using UV light was at -20℃, 24 h, and then raised to 20℃ (5℃/h), 48 h.

Semithin Sect.  (0.8–1.0 μm) and Ultrathin Sect.  (70–90 nm) were cut using a Reichert Ultracut S or Leica EM UC6 (Leica, Vienna, Austria). The semithin sections were collected on slides, and stained with 1% toluidine blue O/Na borax at 60℃, and the images were gotten using Canon EOS 650D connected with Olympus BX40 light microscope. The ultrathin sections were collected using 100 mesh copper grids for morphology and 100 mesh nickel grids for immunolabeling. The sections were stained with 5% uranyl acetate in 50% methanol for 10 min and 0.5% lead citrate for 4 min. Ultrathin sections were observed using a FEI Tecnai G2 Spirit TWIN Transmission Electron Microscope at 80 KV and the images are gotten with Gatan Orius CCD camera.

## Results

### Root tip of *Arabidopsis thaliana*, 0.1%UA + 1%OsO_4_/Acetone, Spurr’s resin embedding

**Fig. 1:**
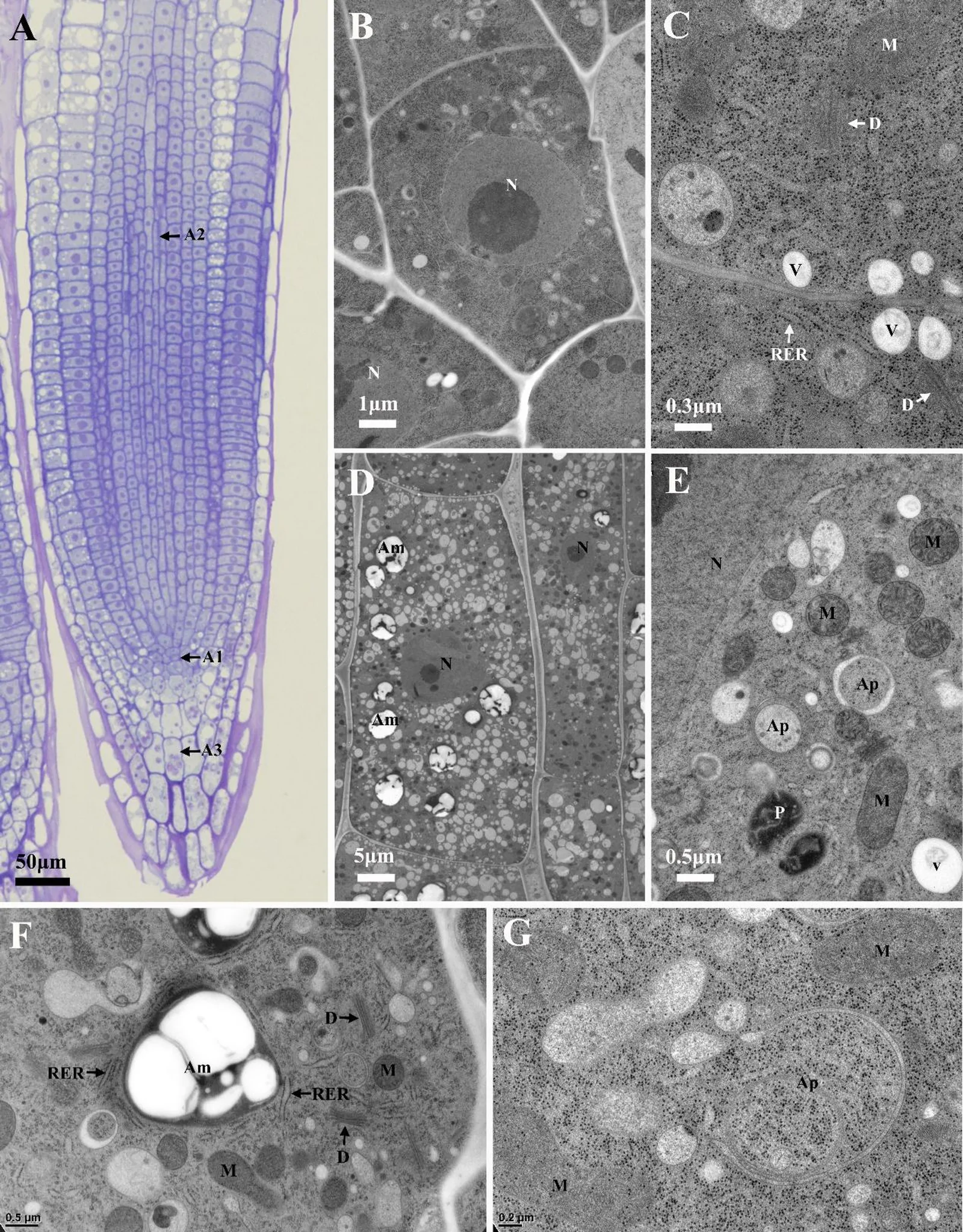
Root tip of *Arabidopsis thaliana*. (**A**) Longitudinal section of root tip showing root cap, meristematic zone, elongation zone and differentiation zone. (**B**) Meristematic cell at A1. (**C**) Meristematic cell showing cell contents. (**D**) Developing tracheary cell at A2. (**E**) Developing tracheary cell showing nucleus, plastids, mitochondria, Golgi bodies, rough ER and autophagosomes. (**F**) Root cap cell at A3 showing amyloplasts, mitochondria, Golgi bodies and rough ER. (**G**) Root cap cell showing autophagosome developing. Am, amyloplast; Ap, autophagosome; D, Golgi body; M, Mitochondrion; N, Nucleus; P, Plastid; RER, rough ER; V, Vacuole

The root tip of *Arabidopsis thaliana* is composed of root cap, meristematic zone, elongation zone and differentiation zone (Fig. 1A), and the latter three regions are overlapping. The ultrastructure is preserved very well in different cell types of root tip. The meristematic cell (Fig. 1B) in quiescent center has a dominant nucleus and various organelles including mitochondria, plastids, Golgi bodies, rough ER and small vacuoles. By enlargement, the cell wall of meristematic cell is thin, and the ribosomes and the lipid bilayer of plasma membrane and small vacuoles are very clear (Fig. 1C).

The developing tracheary cell (Fig. 1D) in elongation zone has the formation of secondary wall. Some amyloplasts and many small vacuoles are present in this cell. The organelles, including plastids, mitochondria, Golgi bodies and rough ER, are clear (Fig. 1E). Some developing stages of autophagosome can be easily found in this cell (Fig. 1E).

The root apical meristem is surrounded by root cap. The large amyloplasts and organelles are present in root cap cell (Fig. 1F). The Golgi bodies are very active to produce vesicles. Autophagy occurs in this cell, and lipid bilayer of phagophore is very clear (Fig. 1G).

### Anthers of *Arabidopsis thaliana*, 0.1%UA + 1%OsO_4_/Acetone, Spurr’s resin embedding

**Fig. 2:**
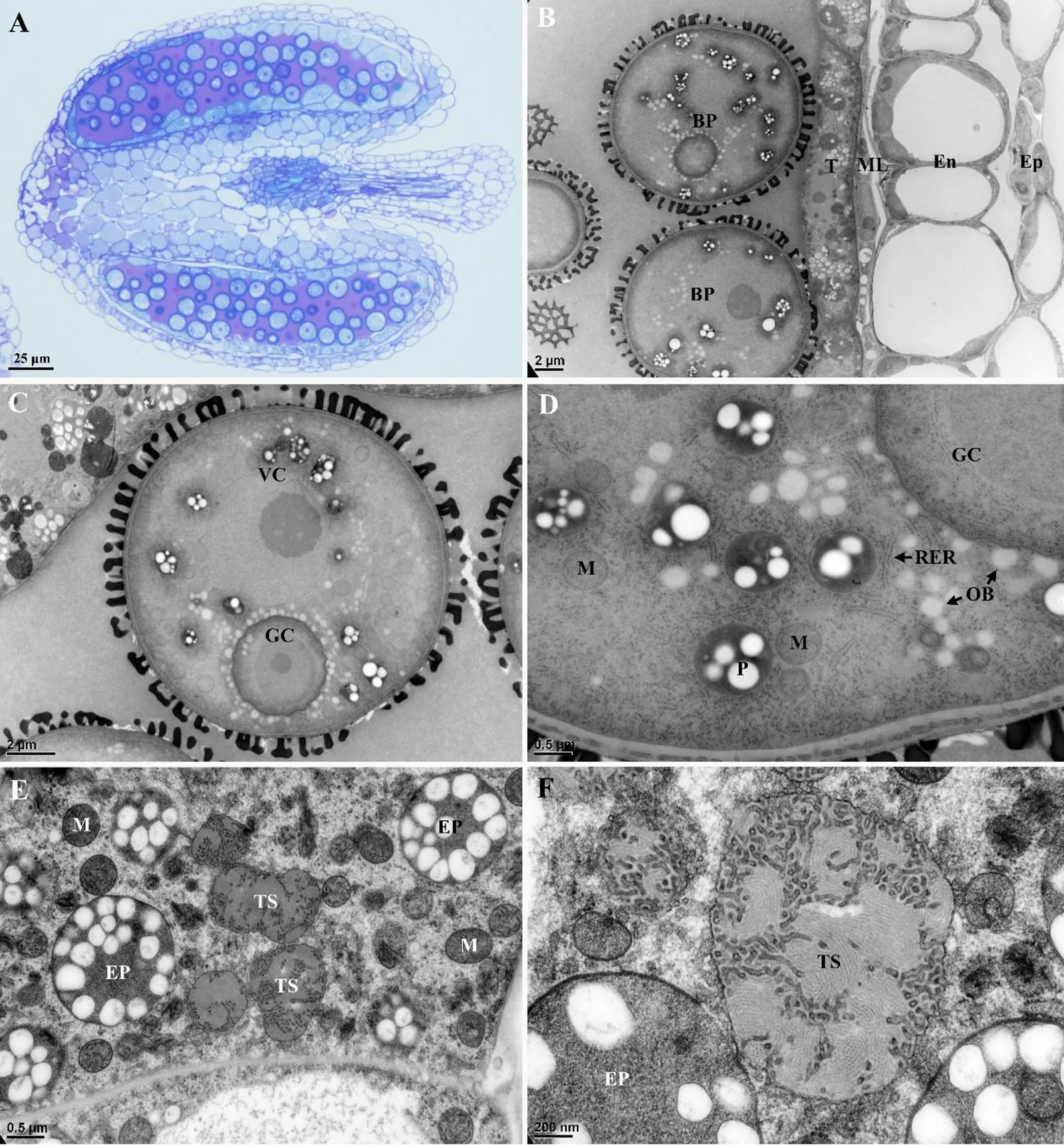
Anther of *Arabidopsis thaliana*. (**A**) Longitudinal section of anther during bicellular pollen stage. (**B**) Bicellular pollens and anther wall consisted of epidermis, endothecium, middle layer and tapetum. (**C**) Bicellular pollen containing vegetative cell and generative cell. (**D**) Many organelles present in the cytoplasm of vegetative cell. (**E**) Some elaioplasts and tapetosomes present in tapetal cell. (**F**) Tapetosomes. BP, Bicellular pollen; En, Endothecium; Ep, Epidermis; EP, Elaioplast; GC, Generative cell; M, Mitochondrion; ML, Middle layer; OB, Oil body; P, Plastid; RER, rough ER; T, Tapetum; TS, Tapetosome; VC, vegetative cell

The anthers during bicellular stage (Fig. 2A) are preserved well by 3 min degas-pumped. The ultrastructure is good in cells of pollens and anther wall. The anther wall is composed of epidermis, endothecium, middle layer and tapetum (Fig. 2B). The middle layer is compressed and collapsed. The epidermis, endothecium, and tapetum are more complete. In thick-walled bicellular pollen (Fig. 2C), the generative cell is enclosed in the vegetative cell. Many lipid bodies are present in the cytoplasm of vegetative cell and surround the generative cell. The various organelles are present in the cytoplasm of vegetative cell (Fig. 2D), including starch-containing plastids, mitochondria, Golgi bodies and rough ER. Many elaioplasts and tapetosomes are present in the cytoplasm of tapetal cell (Fig. 2E). Inner structure of tapetosomes composed of stacks of ER cristae is very clear (Fig. 2F).

### Leaves of *Brachypodium distachyon*, 2%OsO_4_/Acetone, EMBed 812 resin embedding

**Fig. 3:**
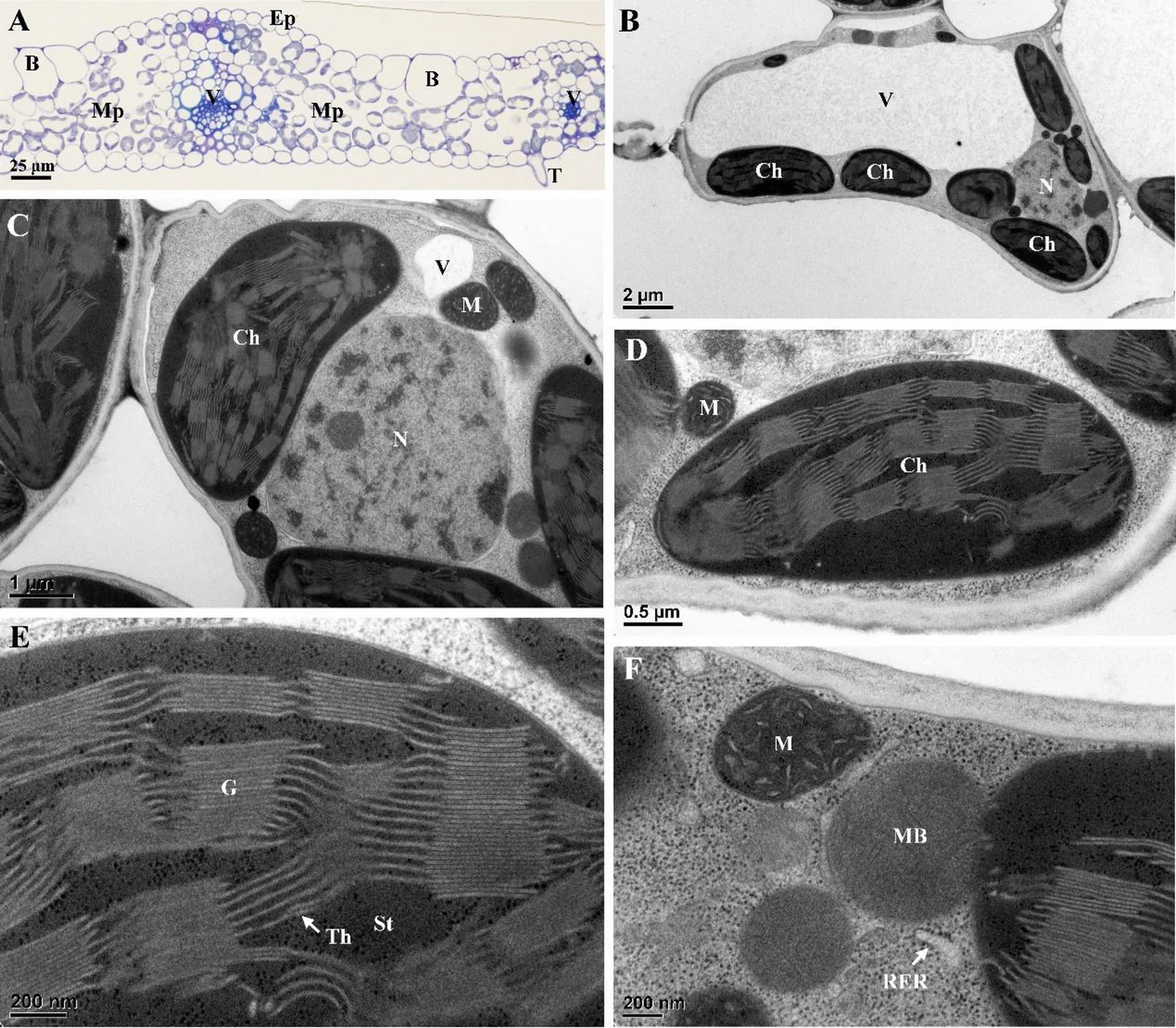
Leaf of *Brachypodium distachyon*. (**A**) Cross section of leaf blade, consisted of epidermis, mesophyll and veins. Bulliform cells in the adaxial epidermis are located between two veins. (**B**) Mesophyll cell, composed of a nucleus, chloroplasts, a large vacuole, and mitochondria. (**C**) The nucleus, chloroplasts, mitochondria, and microbodies are present in mesophyll cell. (**D**) The chloroplast, composed of stroma and thylakoids. (**E**) The chloroplast. Thylakoids are stacked to grana and lipid bilayer of membrane is clear. Many ribosomes are present in stroma. (**F**) Mesophyll cell showing chloroplasts, mitochondria, microbodies, rough ER, and ribosomes. B, Bulliform cell; Ch, Chloroplast; Ep, Epidermis; G, Granum; M, Mitochondrion; MB, Microbody; Mp, Mesophyll; N, Nucleus; RER, rough ER; ST, Stroma; T, Trichome; Th, Thylakoid; V, Vacuole; Ve, Vein

*Brachypodium distachyon* is a C3 grass. The results are good using degas-pumping of 6 min. The thickness of mature leaves is approximately 80 μm. Leaf is composed of epidermis, mesophyll and veins (Fig. 3A). Bulliform cells are present in upper epidermis and between two veins. A mesophyll cell contains a nucleus, a large vacuole and some chloroplasts (Fig. 3B). Besides nucleus and chloroplasts, some mitochondria and microbodies are present in the cytoplasm of mesophyll cell (Fig. 3C). The chloroplast consists of thylakoids and stroma (Fig. 3D). In the chloroplast, many ribosomes are present in stroma and thylakoids are stacked to grana (Fig. 3E). Lipid bilayer of thylakoid membrane is very clear. In the cytoplasm of mesophyll cell, mitochondria have well-developed cristae, and microbodies and rough ER can be identified clearly (Fig. 3F).

### Leaves of *Axonopus compressus*, 2%OsO_4_/Acetone, Spurr’s resin embedding

**Fig. 4:**
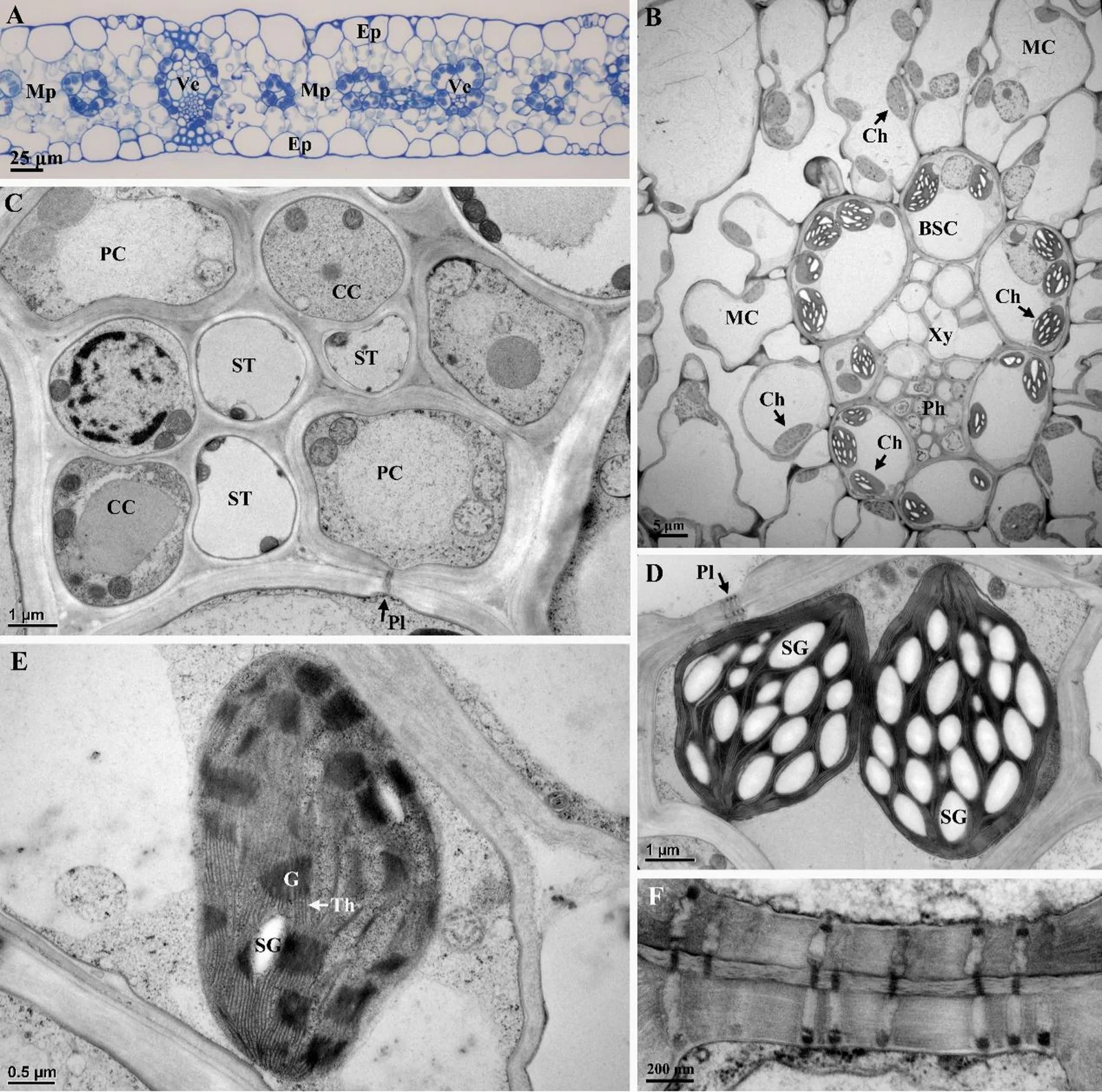
Leaf of *Axonopus compressus*. (**A**) Cross section of leaf blade, consisted of epidermis, mesophyll and veins. (**B**) Vascular tissue, bundle sheath and mesophyll. The chloroplasts are dimorphic. (**C**) Phloem composed of sieve tube members, companion cells and parenchyma cells. (**D**) Bundle sheath cell showing two chloroplasts. (**E**) Mesophyll cell showing one chloroplast. (**F**) Plasmodesmata located in cell wall between two bundle sheath cells. BSC, Bundle sheath cell; CC, Companion cell; Ch, Chloroplast; Ep, Epidermis; G, Granum; MC, Mesophyll cell; Mp, Mesophyll; PC, Parenchyma cell; Ph, Phloem; Pl, Plasmodesma; SG, Starch grain; ST, Sieve tube member; Th: Thylakoid; V, Vacuole; Ve, Vein; Xy, Xylem

*Axonopus compressus* is a C4 grass. The results are good using degas-pumped 8 min. The thickness of mature leaves is approximately 100 μm. Leaf is composed of epidermis, mesophyll and veins (Fig. 4A). The vein is surrounded by the bundle sheath. The chloroplasts are present in mesophyll cells and bundle sheath cells (Fig. 4B). The leaf veins consist of phloem and xylem. The phloem is composed of sieve tube members, companion cells and parenchyma (Fig. 4C). The ultrastructure of all phloem cells is very clear. The cell compartments can be identified easily. The chloroplasts in bundle sheath cells are electron denser than in mesophyll cells (Fig. 4B). The chloroplasts of mesophyll cell contain many thylakoids, stacked to grana, and less starch grains (Fig. 4E). The chloroplasts of bundle sheath cells contain many starch grains and some thylakoids (Fig. 4F). Lipid bilayer of thylakoid membrane is very clear. Many plasmodesmata are present in the cell walls between two bundle sheath cells (Fig. 4F), and between bundle sheath cells and mesophyll cells.

### Leaves of *Plantago asiatica*, 2%OsO_4_/Acetone, spurr’s resin embedding

**Fig. 5:**
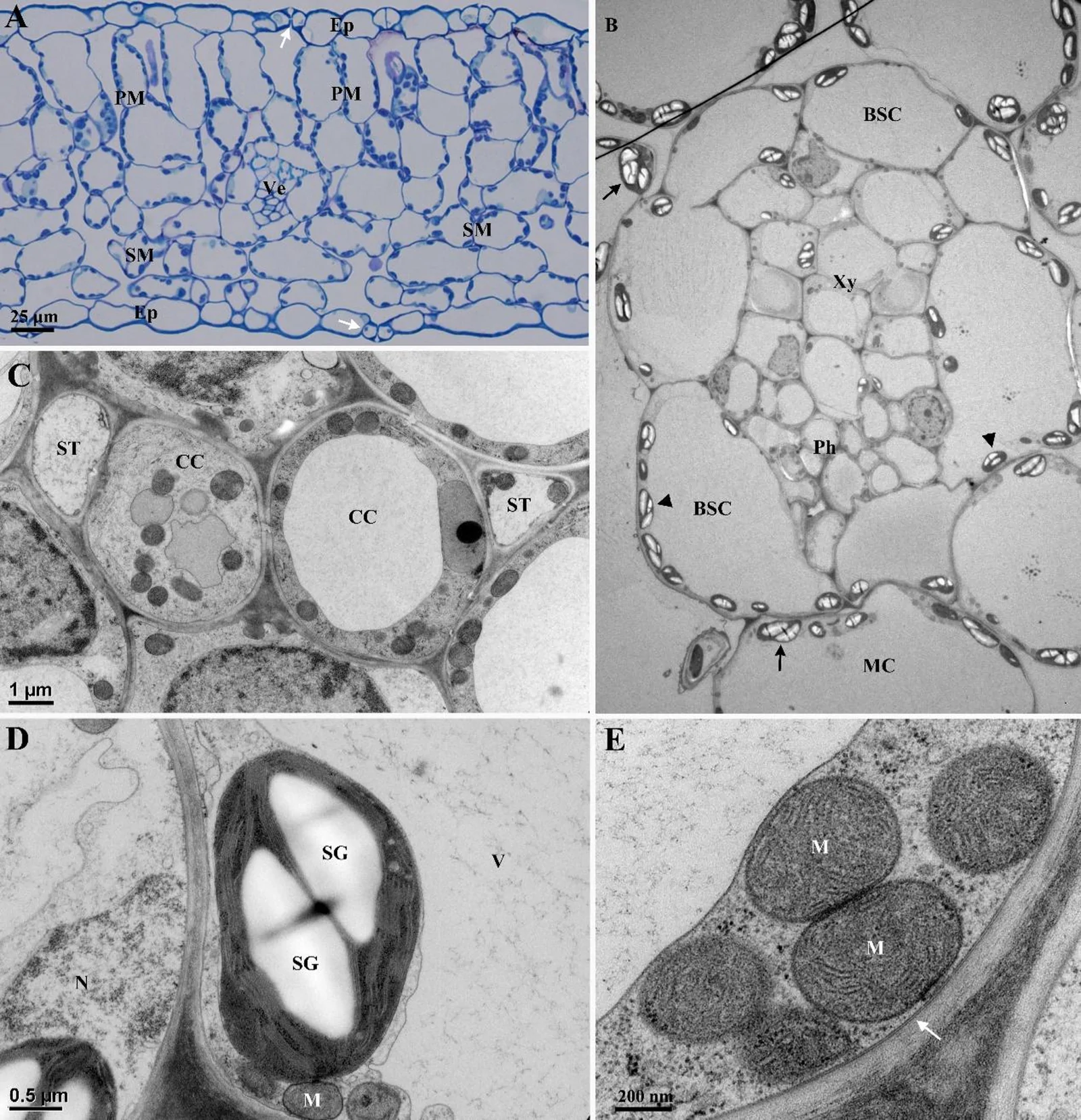
Leaf of *Plantago asiatica*. (**A**) Cross section of leaf blade, consisted of epidermis, mesophyll and veins. Some stomatal complex (arrow) are present in abaxial and adaxial epidermis. Mesophyll can be divided into palisade and spongy tissues. (**B**) The vein, consisted of phloem and xylem, is surrounded by a bundle sheath. Chloroplasts of bundle sheath cells (arrowhead) are smaller than those of mesophyll cells (arrow). (**C**) Phloem showing two sieve tube members and two companion cells. (**D**) Palisade mesophyll cell showing a nucleus, a nucleus, and two mitochondria. Thylakoids and grana are present in chloroplast. (**E**) Spongy mesophyll cell showing mitochondria, plasma membrane (arrow) and tonoplast membrane (arrowhead). Mitochondria have well-developed cristae. BSC, Bundle sheath cell; CC, Companion cell; Ch, Chloroplast; Ep, Epidermis; G, Granum; M, Mitochondrion; MC, Mesophyll cell; PC, Parenchyma cell; Ph, Phloem; PM, Palisade mesophyll; SG, Starch grain; SM, Spongy mesophyll; ST, Sieve tube member; Th. Thylakoid; V, Vacuole; Ve, Vein; Xy, Xylem

*Plantago asiatica* is a campus plant in Taiwan. The results of mature leaves are good using degas-pumped 10 min. The thickness of mature leaves is 180–200 μm. Leaf is composed of epidermis, mesophyll and veins (Fig. 5A). The vein, composed of phloem and xylem, is surrounded by the bundle sheath (Fig. 5B). The chloroplasts containing starch grains are present in mesophyll cells and bundle sheath cells (Fig. 5B). The size of chloroplasts in bundle sheath cells is less than that in mesophyll cells. In phloem, the sieve tube members and the companion cells can be identified easily (Fig. 5C). The organelles of sieve tube members are only left with mitochondria. Companion cells are characterized by the presence of numerous mitochondria. The ultrastructure of all compartments in phloem is very clear. The mesophyll cells contain a large vacuole, a nucleus, many chloroplasts and mitochondria. The chloroplasts of mesophyll cell contain large starch grains (Fig. 5D). Many thylakoids are present in the chloroplast and some stacked to grana. Lipid bilayer of thylakoid membrane is very clear. Lipid bilayers of mitochondrial cristae, plasma membrane and tonoplast membrane are also very clear (Fig. 5E).

### Petals of *Phalaenopsis bellina*, 0.1%UA + 0.2%GA/Ethanol, LR Gold resin embedding

**Fig. 6:**
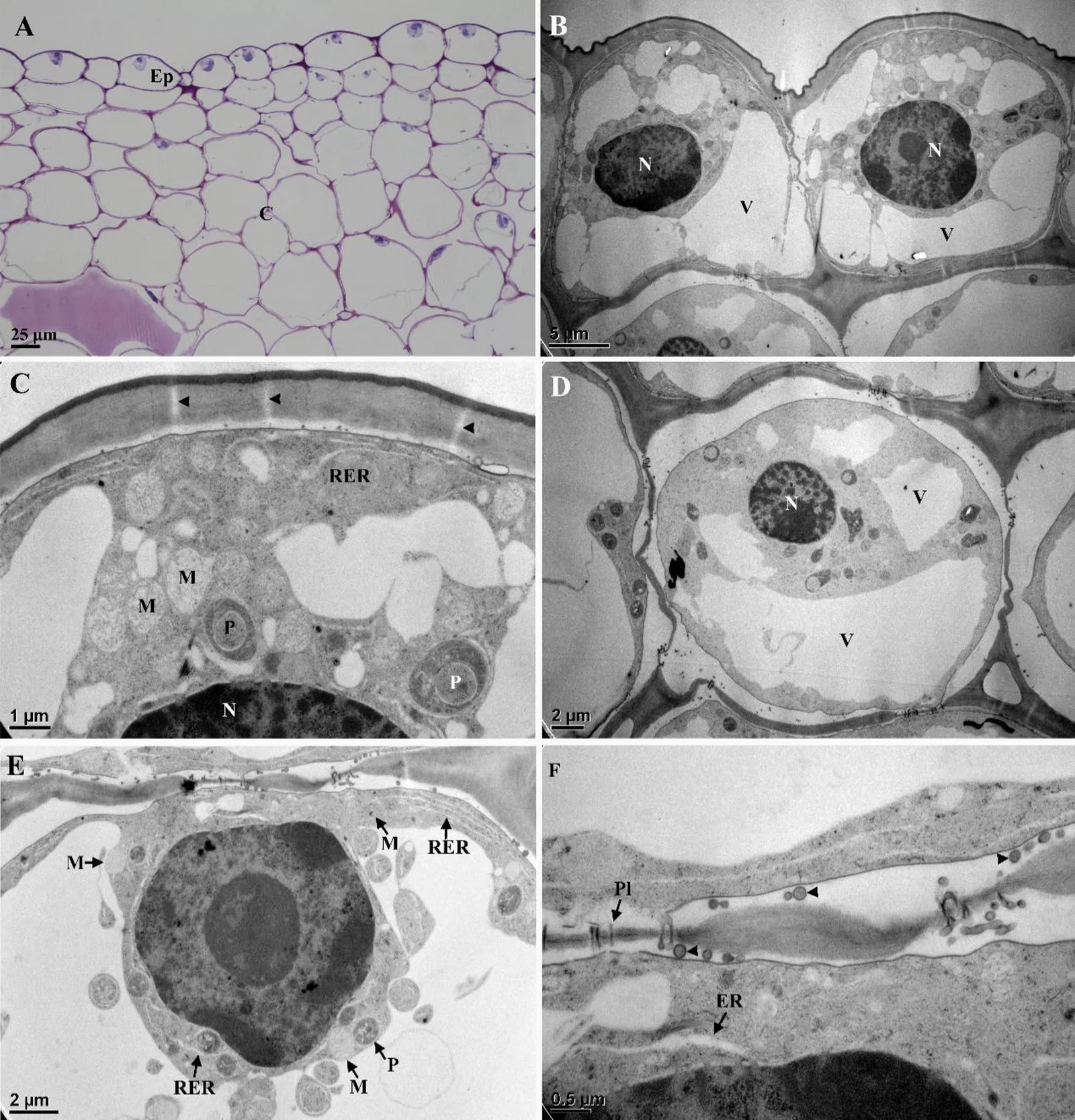
Petals of *Phalaenopsis bellina*. (**A**) Cross section of petal showing epidermis and cortex. (**B**) Adaxial epidermal cells showing cell contents, large nuclei and vacuoles. (**C**) Adaxial epidermal cell showing cell contents. Many organelles are present in cytoplasm. Some electron-transparent channels (arrowhead) are in the cell wall. (**D**) Cortical cells showing cell contents. (**E**) Cortical cells showing nucleus and organelles. (**F**) Cortical cells showing many exocytic vesicles (arrowhead) between plasma membrane and cell wall, and plasmodesmata in cell wall. Ep, Epidermis; C, Cortex; M, Mitochondrion; N, Nucleus; P, Plastid; Pl, Plasmodesma; RER, rough ER; V, Vacuole

*Phalaenopsis bellina* is a scented orchid when blooming. The thickness of petals during blooming first day at basal region is over 600 μm. The results of first day blooming petals are good using degas-pumped 12 min. Petals are consisted of epidermis, cortex and vein (Fig. 6A). The cell volume is occupied by large vacuoles in epidermal and cortical cells. The size of cortical cells become larger towards central region. The abaxial epidermal cells contain large nuclei and some vacuoles (Fig. 6B). The organelles including plastids, mitochondria and rough ER are present in the cytoplasm of epidermal cells (Fig. 6C). Some electron-transparent channels are present in the cell wall towards outer space. The cortical cells also have large nuclei and vacuoles (Fig. 6D). The nucleus has a dominant nucleolus (Fig. 6E). The organelles including plastids, mitochondria and rough ER are present in the cytoplasm of cortical cells. Some rough ER arrange parallel with cell walls. Many exocytic vesicles can be found in the space between plasma membrane and cell walls (Fig. 6F). Plasmodesmata are present in cell wall.

## Discussion

Plant sample preparation of transmission electron microscopy using HPF-FS has the advantage of maintaining good original structure of cells. Therefore, it is a good choice whether it is morphological observation of cell ultrastructure, immunolocalization of specific components, or tomography, which can almost completely replace traditional chemical fixation. However, the good results of HPS-FS are more difficult than the traditional chemical fixation.

Among the works on the preparation of plant samples by the HPF-FS, Hess (2007), Karahara and Kang (2014), Seguí-Simarro (2015) focused on methodology. Many studies on *Arabidopsis thaliana* have been conducted such as pollen development (Otegui and Staehelin, 2004; Yamamoto et al. 2003), leaves (Kirchhoff et al. 2011), root tips (Kiss et al. 1990; Segui-Simarro, et al., 2004; Kang 2010; Park et al. 2013) and shoot tips (Kang 2010; Charuvi et al. 2012). Leaves of rice (Bourett, et al., 1999) and *Lactuca sativa* (Shimoni et al. 2005; Bussi, et al. 2019) were also done.

The results presented by these works seem to be limited to the fine structure of a local area or a local cell, rather than a larger area, such as a root tip or a young leaf. This article pursues a whole section of a root (1 mm long) or a small mature leaf (1 mm x 1 mm), which covers a wide range of cells and their internal fine structures. In order to achieve this goal, both high-pressure freezing and freeze substitution must be considered.

Two aspects for high-pressure freezing of plants need to be considered, the degree of intracellular vacuolation and the amount of air in the intercellular space. The freezing efficiency is positively correlated with the concentration of the solute, so the higher the cell composition density of the target, the better the freezing effect. Compared with animal cells, except meristems, plant cells all contain vacuoles of different sizes and proportions, especially mature cells. The vacuoles occupy most of the cell volume, and the vacuoles are composed of water and water-soluble substances. Therefore, the cell composition density of plant cells is much lower than that of animal cells, and the freezing effect is poor. High vacuolated plant cells, such as tree leaves, are difficult to freeze, because the cell volume is occupied almost entirely by vacuoles in which the high-water content is present (Karahara and Kang, 2014).

When samples are frozen under high pressure, a cryoprotectant is usually added to fill and remove the air in the carrier cavity inside and outside the sample, so as not to affect the heat conduction, because air is the biggest obstacle to freeze. The more air it contains, the harder it is to freeze. Therefore, degas is an important issue for plant freezing, especially mature plant leaves. What kind of plant tissues need to degas during high pressure freezing? The air content in the plant tissue is sufficient to affect the progress of freezing and cause the freezing effect to be poor.

In plants, intercellular spaces play a critical role in water transport (e.g., in roots) and gas exchange (e.g., in stems, leaves, and anthers). The volume fraction of intercellular spaces is minimal in the root tip area but increases slightly in mature regions, where these spaces are filled with either aqueous solutions or air, depending on the tissue type. The cells of shoot apex are arranged extremely tightly, and the proportion of gas filled is very small, so there is no need to degas. The same applies to young anthers and young leaves. The mature leaves contain the high ratio of intercellular spaces containing air, thus the degas action is need. The more completely the degas is frozen, the better the effect.

There are two important factors in the progress of degas, which cryoprotectant is used and what method is used? 1-Hexadecene, a paraffin oil with low surface tension, is the most commonly used as cryoprotectant/filler for plant samples, such as leaves, sepals and petals. It can permeate into intercellular air spaces and replace the air (Morphew 2000).

By using HPF-FS technology to prepare biological samples, the excellent results for animal cells (Figure S1 A-B) or tissues (Figure S1 C-F) and microorganisms (Figure S2) have been yielded, with a complete and clear ultrastructure, and many have been published (Chang et al. 2013; Sheu et al. 2018, 2023; Huang, et., 2019; Fu et al. 2020). For plants, when using 1-hexadecene as a cryoprotectant/filler, the roots of *Arabidopsis thaliana* could produce excellent results (Fig. 1). However, the anthers of *Arabidopsis thaliana* in later stages of development often yielded poor results without degassing, with severe loss of intracellular contents (Figure S3 A-D). Degassing using a syringe to create a micro-vacuum resulted in better results (Figure S3 E-F) than without degassing. Before using a vacuum desiccator for degassing, a syringe to create a micro-vacuum was used. When the samples were mature leaves, as *Arabidopsis thaliana*, the results were mostly poor, with unclear fine structures (Figure S4 A-E); only a few results were acceptable (Figure S4 F).

After failing to identify the aroma release mechanism of orchids using chemical fixation for immunolocalization, the aroma-releasing parts of the sepals or petals with HPF-FS and using LR Gold resin as the embedding agent were tried, and aiming to clearly visualize exocytosis. First, in the samples without degas treatment, the cortical cells ruptured (Figure S5 A), and the organelles were unidentifiable; while with degas treatment, although the cells remained intact (Figure S5 B), the intracellular contents were destroyed by ice crystals, and the fine structure was unclear (Figures S5 C-D).

After several failures, the samples placed in a vacuum desiccator for degassing was tried as undergoing chemical fixation. The results were that the cells remained intact (Figure S5 E), and the organelles became identifiable (Figure S5 F). Next, the vacuum level was controlled and performed degassing treatment for different times. The 12-minute treatment showed the best results, maintaining cell integrity and revealing the fine structure (Fig. 6). After recognizing the relationship between degassing degree and leaf thickness, leaves of *Brachypodium distachyon* (Figure S6 A) were subjected to degassing for different durations, with 6 min yielding the best results (Fig. 3). Subsequently, leaves of *Arabidopsis thaliana* (Figure S6 B), *Axonopus compressus* (Figure S6 C), *Oryza sativa* (Figure S6 D), *Plantago asiatica* (Figure S6 E), and petals of *Phalaenopsis violacea* (Figure S7 B) and *Phalaenopsis bellina* (Figure S7 A) were subjected to the same treatment to determine the optimal degassing time for freezing. The same method yielded better results when applied to anthers of *Arabidopsis thaliana* (Fig. 2). The results shown are the best obtained to date. Table 1 presents our experimental results, demonstrating a positive correlation between leaf thickness and the optimal degassing duration for each plant. The real influencing factor is actually the air content (volume) in the intercellular spaces, which corresponds exactly to the leaf thickness. This indicates that using a vacuum desiccator to control the vacuum degree during degassing is a good method for high-pressure freezing, not only achieving good freezing results but also providing a quantitative basis.

**Table 1:** Our experimental results showing a positive correlation between leaf thickness and the optimal degassing time for each plant

Plant	Thickness of leaf or petal	Degassing Time
*Brachypodium distachyon*	80 μm	6 min
*Arabidopsis thaliana*	80 μm	6 min
*Axonopus compressus*	100 μm	7 min
*Oryza sativa*	120 μm	8 min
*Plantago asiatica*	200 μm	10 min
*Phalaenopsis violacea*	600 μm	12 min
*Phalaenopsis bellina*	600 μm	12 min

Freeze substitution involves placing the frozen sample in an organic solvent, allowing the water of crystallization to slowly precipitate out at low temperatures. Therefore, the general procedure is to remain below − 70 °C (commonly − 85 °C or -90 °C) for an appropriate time. However, 1-Hexadecene is used as a cryoprotectant, which requires thawing to above − 68 °C (Morph, 2000) to dissolve when frozen. In other words, if the sample is coated with 1-Hexadecene, it needs to be thawed to above − 68 °C before it comes into contact with the replacement solution and freezing replacement can occur. Therefore, after placing the frozen sample in the replacement solution, the frozen 1-Hexadecene coating the sample surface must be removed to allow freezing replacement to proceed at -85 °C or -90 °C. If 1-Hexadecene has seeped into the intercellular spaces, the time the frozen sample remains below − 70 °C in the substitution solution and the thawing time from − 70 °C to -60 °C should be extended to complete the freeze substitution. For morphological observation, a 2% OsO_4_ or 0.1% UA + 2% OsO_4_ /Acetone substitution solution and Spurr’s resin (Spurr 1969) or EMBed 812 resin (Luft 1961) as the embedding agent are used typically. For immunolabeling, a 0.1% UA + 0.2% GA/EtOH substitution solution and LR Gold resin (Thorpe 1999) as the embedding medium are commonly used.

In leaf of *Arabidopsis thaliana*, the thylakoid membrane is negatively stained and difficult to identify under normal protocol of freeze substitution; after washing away OsO_4_ at -20 °C, the thylakoid membrane of chloroplasts is well contrasted (Austin II 2014). However, the negatively-stained thylakoids are not found in this research.

## Conclusions

During high pressure freezing, 1-Hexadecene is an ideal cryoprotectant for plant tissues, whether young or mature. In the mature plant leaves, degassing by pumping in vacuum desiccator is a good method to reduce gas interference with the freezing process and getting the good freezing results. The results in leaves of *Brachypodium distachyon*, *Axonopus compressus*, *Plantago asiatica*, and petals of *Phalaenopsis bellina* are checked, and the optimal degassing duration for each plant is a positive correlation with leaf or petal thickness. After freeze substitution, the ultrastructure is well preserved and it has better antigenicity.

## Supplementary Information

Below is the link to the electronic supplementary material.

Supplementary Material 1

## Data Availability

All images generated during this study are included in this article.
