# Supplementary material for: Sample preparation of some plant tissues for transmission electron microscopy using high pressure freezing and freeze substitution
Source: Bot Stud. 2026 Oct 15;67:13. doi: 10.1186/s40529-026-00499-x (PMC13672732; doi:10.1186/s40529-026-00499-x)
Supplement: Supplementary file 1 [file 40529_2026_499_MOESM1_ESM.docx]

**Supplementary Information**

Figure S1: Animal cells and tissues using HPF-FS. A. Lunger cancer cell with liposomes (arrowhead). B. Cell contents of lunger cancer cell with liposomes (arrowhead). C. Eye from stem cell NTA 108. D. Cell contents of eye cell. E. Brown adipose tissue of mouse. F. Brown adipose tissue of mouse showing cell contents. LB, Lipid body; M, Mitochondrion; N, Nucleus; RER, rough ER.

Figure S2. Microbials using HPF-FS. A. ***Simiduia agarivorans*** (SA1). B. ***Simiduia agarivorans*** (SA1). C. ***Pseudomonas*** sp. H9. D. ***Pseudomonas*** sp. H9. E. ***Galdieria maxima***. F. ***Galdieria maxima*** showing phycobilisomes (arrowhead) binding with thylakoid membrane. Ch, Chloroplast; M, Mitochondrion; N, Nucleus; Th, Thylakoid.

Figure S3. Anthers of ***Arabidopsis thaliana*** using HPF-FS. A-D. No degas in 1-hexadecene. E-F. Degas in 1-hexadecene using vacuum created by pumping air from a syringe. A. Anther during vacuolated microspore stage showing results of poor freezing effect. B. A vacuolated microspore of poor freezing effect showing loss of cell contents.

C. Anther during mature microspore stage showing results of poor freezing effect. D. A mature pollen grain of poor freezing effect showing loss of cell contents. E. A vacuolated microspore of good freezing effect showing clear ultrastructure. F. A mature pollen grain of good freezing effect showing clear ultrastructure. Am, Amyloplast; M, Mitochondrion; N, Nucleus; S, Sperm; T, Tapetum; V, vacuole. VN, Vegetative nucleus.

Figure S4. Leaves of ***Arabidopsis thaliana*** using HPF-FS. A-B. No degas in 1-hexadecene. C-F. Degas in 1-hexadecene using vacuum created by pumping air from a syringe. Ch, Chloroplast, G, Granum; SG, Starch grain; Th, Thylakoid; Ve, Vein.

Figure S5. Petal of *Phalaenopsis* sp. using 1-hexadecene and HPF-FS. A-F. *P. bellina*. G-H. *P. violacea*. A. No degas. B-D. Degas using vacuum created by pumping air from a syringe. E-H. Degas in a vacuum desiccator and pumping. A. LM image of cross section showing cell broken (arrowhead). B. LM image of cross section. C-D. Organelles cannot be identified in cytoplasm. E. LM image of cross section. F. Organelles can be identified in cytoplasm of epidermal cell. G. Organelles can be identified and clear in cytoplasm of epidermal cell. H. Organelles can be identified and clear in cytoplasm of cortical cell.

Figure S6. A-E. LM images using HPF-FS. Cross sections of leaves showing structure and thickness. A. *Brachypodium distachyon*, 80um. B. *Arabidopsis thaliana*, 80um. C. *Axonopus compressus*, 100um. D. *Oryza sativa*, 120um. E. *Plantago asiatica*, 200um.

Figure S7. A-B. LM images. Cross sections of petals showing structure and thickness. A. *Phalaenopsis violacea*, 600um. Using chemical fixation. B. *Phalaenopsis bellina*, showing a half thickness 300um. Using HPF-FS.
